# Association between early detected heart failure stages and future cardiovascular and non-cardiovascular events in the elderly (Copenhagen Heart Failure Risk Study)

**DOI:** 10.1186/s12877-022-02875-1

**Published:** 2022-03-21

**Authors:** Saaima Parveen, Bochra Zareini, Anojhaan Arulmurugananthavadivel, Caroline Kistorp, Jens Faber, Lars Køber, Christian Hassager, Tor Biering Sørensen, Charlotte Andersson, Deewa Zahir, Kasper Iversen, Emil Wolsk, Gunnar Gislason, Freja Gaborit, Morten Schou

**Affiliations:** 1 Department of Cardiology, Copenhagen University Hospital Herlev and Gentofte, Copenhagen, Denmark; 2 Department of Endocrinology, Rigshospitalet, Copenhagen University Hospital, Copenhagen, Denmark; 3 Department of Clinical Medicine, University of Copenhagen, Copenhagen, Denmark; 4 Department of Endocrinology, Copenhagen University Hospital Herlev and Gentofte, Copenhagen, Denmark; 5 Department of Cardiology, Rigshospitalet, Copenhagen University Hospital, Copenhagen, Denmark; 6 Department of Medicine, Section of Cardiovascular Medicine, Boston Medical Center, Boston University School of Medicine, Boston, MA USA; 7 The Danish Heart Foundation, Copenhagen, Denmark

**Keywords:** Heart failure, Heart failure stages, Population attributable risk, Comorbidity, All-cause mortality

## Abstract

**Background:**

Early stages of heart failure (HF) are associated with an increased risk of hospitalization and increased mortality, however the course of progression and the impact of non-cardiovascular comorbidities on adverse events in elderly high-risk patients are unknown.

**Aim:**

To examine the risk of future cardiovascular (CV) and non-CV events in early stages of HF in a cohort of elderly patients (age ≥ 60 with ≥ 1 risk factor for HF and without known or clinically suspected HF).

**Methods:**

A total of 400 patients (American Heart Association HF stage A: *N* = 177; stage B: *N* = 150; stage C: *N* = 73) from the Copenhagen Heart Failure Risk Study were identified and followed for the main composite outcome of a HF hospitalization (HFH), ischemic heart disease (IHD), stroke, and all-cause death, recorded within the Danish nationwide registries. Non-CV hospitalization was a secondary outcome. Absolute risk was calculated by the Aalen-Johansen estimator.

**Results:**

The median follow-up time was 3.3 years, total number of events were 83, and the 3-year risk (95% confidence interval) of the main outcome was 12.8% (7.8–17.9), 22.8% (16.1–29.6) and 31.8% (21.0–42.6) for patients with stage A, B, and C, respectively. 1.1% (0.0–2.7), 3.4% (1.0–6.3) and 10.0% (2.8–16.3) experienced HFH as their first event, whereas 37.3% (30.2–44.4), 49.7% (41.6–57.8) and 54.8% (43.4–66.2) were admitted for non-CV causes as their first event.

**Conclusion:**

The risk of HFH, IHD, stroke and all-cause death increased with severity of HF stage, and 10% of patients with undiagnosed HF stage C were admitted for HF within 3 years. However, the risk of non-CV hospitalizations was greater compared to the risk of experiencing HFH.

**Supplementary Information:**

The online version contains supplementary material available at 10.1186/s12877-022-02875-1.

## Introduction

The natural course of progression from The American College of Cardiology (ACC)/American Heart Association (AHA) heart failure (HF) stage A to D is poorly elucidated [1–3] and the intersection with non-cardiovascular (CV) comorbidities are even less well understood. Asymptomatic HF is defined as stage A (risk factors for HF) or B (abnormal echocardiography), whereas symptomatic HF is defined as stage C (abnormal echocardiography and symptoms of HF) or D (refractory symptoms despite optimal medical treatment). Three larger observational studies have evaluated the association between HF stages and risk of future CV events, but none of them have focused on the interplay with non-CV comorbidities [1–3]. This interaction may have particular interest since HF with preserved ejection fraction (LVEF) is prevalent in the community [1–4] and progression of non-CV comorbidities may dominate in this group of HF patients [5, 6]. Therefore, the development of comorbidities instead of an increased burden of HF over time may contribute to the lack of effect of neurohormonal blockade [7] and modulation [8] on the progression of HF in patients with HF and preserved LVEF, but more data on this topic is needed.

Therefore, in a cohort of elderly patients with HF stage A-C [4], the 3-year risk of death, overt HF, ischemic events, and hospitalization for a non-CV cause was evaluated. In addition, the population attributable risk (PAR) of demographic, cardiac, and extra-cardiac disease burden associated with the risk of the primary outcome was calculated.

## Methods

### Study design and study population

Patients were studied from the Copenhagen Heart Failure Risk Study, a prospective cohort study [4]. Patients were consecutively recruited from December 2014 to June 2016 from the out-patient clinics at the Departments of Cardiology, Diabetology, and Nephrology at the Herlev and Gentofte University Hospital, Copenhagen, Denmark, as a part of a clinical study to examine the prevalence of HF stages in a high-risk elderly population. The rationale, design, and results from the study have been published previously [4]. Inclusion and exclusion criteria are presented in supplementary table S1. Demographic data (age, height, weight, and gender), and data from echocardiography, 12-lead electrocardiogram, venous blood samples, medical history, and medication were obtained during a single visit and were available for statistical analysis. Patients had completed the Minnesota Living with Heart Failure Questionnaire. All patients provided written informed consent for their participation in the study*.*

### Definition of HF stages

The present study and the classification HF stages were defined prior the ACC/AHA and ESC classification of HF stage A, B, and C: HF stage A was defined as the presence of risk factors for HF, with normal echocardiography, and without signs or symptoms of HF [4]. HF stage B was defined as a structural heart disease but without signs or symptoms of HF. HF stage C was defined as the presence of an abnormal echocardiography and past or current symptoms of HF [9]. An abnormal echocardiography was defined according to European Society of Cardiology guidelines (supplementary table S2) [10, 11]. Patients without structural heart disease reporting symptoms were classified as HF stage A and the symptoms were considered non-cardiac, and patients with structural heart disease and normal natriuretic peptides reporting symptoms were classified as HF stage B according to the prespecified definitions in the study [4].

### Data sources

To include information on outcomes we used the Danish nationwide healthcare registries. Each permanent resident in Denmark has a unique personal identification number, enabling linkage on an individual level between registries. All patients included in the study were permanent residents, enabling complete follow-up of all patients. Data was obtained from three administrative registries through Statistics Denmark. The Danish National Patient Registry contains information from 1977 onwards on all hospitalized patients. Each hospital contact is registered with a primary diagnosis and up to several contributing secondary diagnoses at discharge according to the 10^th^ (ICD-10) revision of the International Classification of Diseases. Surgical procedures are registered and coded since 1996 and onwards according to the Nordic Medico‐Statistical Committee Classification of Surgical Procedures. The Causes of Death register contains information on the time of death, causes, and age at the time of death from 1970 onwards and The Danish Civil Registry provides information about age, gender, and birth date since 1968.

### Definition of outcomes—HF admission, stroke, IHD, death, and non-CV admission

All outcomes were assessed through Statistics Denmark. The main outcome was a composite of first HF hospitalization (HFH), ischemic heart disease (IHD), stroke, and all-cause death. Patients were followed until emigration, event, death, or end of the study period (December 31, 2018).

First time HFH was defined as the first overnight hospital stay with either a primary or secondary discharge diagnosis of HF (ICD-10: I110, I130, I132, I42, I426-29, I50). Patients with a reduced LVEF in the study were referred to the public outpatient HF clinic, and this was not considered as an endpoint. IHD was defined as the first hospital contact due to IHD (ICD-10: I20-I22, including primary, secondary diagnoses as well as admission and outpatient contacts), first-time procedure codes for percutaneous coronary intervention, or first-time coronary artery bypass grafting. For further detail of the definition of outcomes see supplementary table S3.

Stroke was defined as the first hospital contact due to stroke (ICD-10: I60-I64). Death was defined as death from all causes. To investigate the burden and causes of non-CV hospitalizations, non-CV hospitalizations were defined as the first overnight hospital stay since inclusion with a primary non-CV discharge diagnosis code (ICD-10: other than I01-99). Hospitalizations that could not be categorized properly registered with referral codes or codes of signs and symptoms compromised their own category. The positive predictive value (PPV) the ICD-10 codes and procedure codes used in this study has previously been estimated and validated [12–14].

### Statistical analysis

Baseline characteristics were presented as counts and percentage (%) for categorical values and continuous variables were presented as means with standard deviation (SD) if normally distributed or as medians with interquartile range with first and third quartiles (Q1-Q3) if not normally distributed. The difference between numerical variables was estimated by the analysis of variance (ANOVA) test or Wilcoxon rank-sum test. The difference among categorical variables was estimated by the Chi-squared test. To investigate the risk of the composite outcome of first-time HFH, IHD, stroke, and all-cause death, we estimated the incidence by the Kaplan–Meier estimator and reported the absolute 1-year and 3-year risk of the composite outcome. The risk of the individual outcomes was estimated by the Aalen-Johansen estimator with all-cause death as competing risk. Incidence of CV and non-CV hospitalizations were estimated with the Aalen-Johansen estimator with all-cause death as competing risk and presented as stacked cumulative plots stratified for each HF stage. A multivariable Cox proportional hazards analysis was performed to compare hazard ratios of the composite outcome according to HF stage with HF stage A as the reference and was further adjusted for age and sex. The contribution of comorbidities to the risk of adverse CV and non-CV outcomes was further investigated by using the Meta-Analysis Global Group in Chronic Heart Failure (MAGGIC) risk score. The MAGGIC model derived an optimal model for predicting mortality in HF patients, with 13 variables identified as being highly significant, and a risk score created using model estimates of the predictive strength of each variable. A higher score was associated with increased risk of death (www.heartfailurerisk.org). The MAGGIC risk score was subdivided into cardiac (LVEF, New York Heart Association class, systolic blood pressure, time since HF diagnosis, HF medication use), extra-cardiac (body mass index, creatinine, diabetes mellitus, chronic obstructive pulmonary disease, smoker) and demographic (age, gender) categories and subscores representing the burden of each component and was calculated individually for all patients [5, 15]. PAR for each component to the main composite outcome of HFH, IHD, stroke, and all-cause death were estimated using the Cox proportional hazards model [5]. The PAR was summarized as a percentage (± 95% confidence intervals) and estimate the hypothetical reduction of incidence rates if all patients had no burden of the specific risk factor (e.g. cardiac disease burden = 0). Attributable risks may add up to greater than 100%, and combined attributable risks are calculated by multiplication. For example, attributable risks of 50% and 60% would produce a combined attributable risk of 1 − (1 − 0.50) × (1 − 0.60) = 80%. Because attributable risks are multiplicative, they are displayed graphically on a log scale so that relative importance can be seen visually. Non-significant negative PAR estimates were considered as 0 [5]. Analyses were conducted using the SAS, R [16], the Prodlim R package [17], and STATA statistical software.

### Ethics

The Copenhagen Heart Failure Risk Study was approved by the Ethical Region of The Capital Region (H-3–2014-016) and conducted according to the Declaration of Helsinki.

## Results

### Baseline characteristics

A total of 400 patients were eligible for follow-up with 177 patients (44%) categorized as stage A, 150 patients (38%) categorized as stage B, and 73 patients (18%) categorized as stage C. Median follow-up was 3,3 years (Q1-Q3: 3;3.7) with no patients lost to follow-up. Baseline characteristics are presented in Table 1. The median age in the study population was 72 years (Q1-Q3: 60;97) with patients in stages B and C being older than those in stage A. Males were slightly overrepresented in the cohort (51.5% vs. 48.5%). The number of risk factors did not differ between HF stages and median plasma concentrations of the cardiac biomarker N-terminal pro-brain natriuretic peptide (NT-proBNP) increased with higher HF-stage (stage A 132.5 ng/L; stage B 275.5 ng/L; stage C 400.0 ng/L, P < 0.001). Mean LVEF was above 50% in all HF stages (LVEF stage A 64. %, SD 7.6; stage B 59%, SD 11.1; stage C 60%, SD 10.7).

**Table 1:** Baseline characteristics

Variable	HF stage A (*n* = 177)	HF stage B (*n* = 150)	HF stage C (*n* = 73)	Total (*n* = 400)	*p*-value
Age, years	69 (60, 90)	75 (60, 97)	74 (61, 94)	72 (60, 97)	< 0.001
Female sex, n (%)	78 (44.1)	74 (49.3)	42 (57.5)	194 (48.5)	0.053
Male sex, n (%)	99 (55.9)	76 (50.7)	31 (42.5)	206 (51.5)	0.148
BMI, kg/m^2^	27.1 (18.2, 44.8)	27.2 (17.1, 41.1)	28.3 (18.0, 50.1)	27.3 (17.1, 50.1)	0.156
BMI ≥ 30, kg/m^2^ (%)	52 (29.4)	39 (26.0)	28 (38.4)	119 (29.8)	0.165
Systolic blood pressure, mm Hg	134 (98, 178)	140 (99, 188)	137 (98, 188)	137 (98, 188)	0.200
Diastolic blood pressure, mm Hg	81 (58, 110)	78 (56, 103)	77 (54, 115)	79 (54, 115)	0.039
HR, beats/min	69 (44, 119)	68.5 (42, 123)	69 (45, 116)	69 (42, 123)	0.941
LV Hypertrophy ECG, n(%)	2 (1.1)	4 (2.7)	1 (1.4)	7 (1.8)	0.552
NYHA class I, n (%)	117 (66.1)	98 (65.3)	20 (27.4)	235 (58.8)	< 0.001
NYHA class II, n (%)	55 (31.1)	45 (30.0)	45 (61.6)	145 (36.2)	< 0.001
NYHA class III, n (%)	5 (2.8)	7 (4.7)	8 (11.0)	20 (5.0)	0.027
Smoking active, n (%)	29 (16.4)	12 (8.0)	7 (9.6)	48 (12.0)	
Smoking never, n (%)	57 (32.2)	69 (46.0)	25 (34.2)	151 (37.8)	
Smoking former, n (%)	91 (51.4)	69 (46.0)	41 (56.2)	201 (50.2)	0.031
MLHFQ	11 (0, 74)	8 (0, 72)	25 (6, 80)	12 (0, 80)	< 0.001
Hypertension, n (%)	145 (81.9)	120 (80.0)	63 (86.3)	328 (82.0)	0.516
Ischemic heart disease, n (%)	37 (20.9)	41 (27.3)	20 (27.4)	98 (24.5)	0.330
Atrial fibrillation, n (%)	40 (22.6)	48 (32.0)	31 (42.5)	119 (29.8)	0.006
Diabetes, n (%)	69 (39.0)	51 (34.0)	23 (31.5)	143 (35.8)	0.454
Chronic kidney disease, n (%)	30 (16.9)	23 (15.3)	10 (13.7)	63 (15.8)	0.801
Apoplexia cerebri, n (%)	19 (10.7)	23 (15.3)	6 (8.2)	48 (12.0)	0.242
Mild COPD or asthma, n (%)	18 (10.2)	6 (4.0)	10 (13.7)	34 (8.5)	0.029
Number of risk factors, n	2 (1, 4)	2 (1, 5)	2 (1, 4)	2 (1, 5)	0.212
More than two risk factors, n (%)	42 (23.7)	44 (29.3)	17 (23.3)	103 (25.8)	0.446
ACE inhibitor, n (%)	49 (27.7)	45 (30.0)	11 (15.1)	105 (26.2)	0.050
Angiotensin receptor antagonist, n (%)	59 (33.3)	51 (34.0)	29 (39.7)	139 (34.8)	0.609
Aldosteron antagonist, n (%)	0 (0.0)	3 (2.0)	2 (2.7)	5 (1.2)	0.120
Calcium antagonist, n (%)	48 (27.1)	50 (33.3)	27 (37.0)	125 (31.2)	0.243
Beta blocker, n (%)	71 (40.1)	75 (50.0)	41 (56.2)	187 (46.8)	0.041
Loop diuretics, n (%)	16 (9.0)	15 (10.0)	29 (39.7)	60 (15.0)	< 0.001
Thiazide, n (%)	62 (35.0)	49 (32.7)	14 (19.2)	125 (31.2)	0.044
Statins, n (%)	115 (65.0)	90 (60.0)	42 (57.5)	247 (61.8)	0.467
Per oral antidiabetics, n (%)	57 (32.2)	35 (23.3)	19 (26.0)	111 (27.8)	0.190
Insulin, n (%)	34 (19.2)	22 (14.7)	9 (12.3)	65 (16.2)	0.326
hsCRP, mg/L	0 (0, 77)	0 (0, 184)	0 (0, 80)	0 (0, 184)	0.099
eGFR, mL/min/1.73m^2^	76 (14, 108)	65.5 (14, 102)	65 (21, 107)	70 (14, 108)	0.003
NT-proBNP, ng/L	132.5 (23, 4,690)	275.5 (10, 10,000)	400 (25, 22,500)	203 (10, 22,500)	< 0.001
LVEF (%)	62.9 (7.6)	57.8 (11.1)	60.0 (10.7)	60.4 (9.9)	0.010
LVEF < 40 (%)	0 (0.0)	8 (5.3)	3 (4.1)	11 (2.8)	0.010
LVmass index	71.3 (31.2, 115.3)	80.3 (34.4, 146.8)	84 (36.8, 213.9)	76.1 (31.2, 213.9)	< 0.001
E/e’ septal	10.5 (5.2, 14.9)	13.1 (6.0, 42.9)	13.2 (6.2, 31.1)	11.5 (5.2, 42.9)	< 0.001
E/e’ lateral	8.2 (3.1, 12.8)	9.6 (4.3, 35.1)	10.4 (4.0, 27.4)	8.6 (3.1, 35.1)	< 0.001
LAvol index, mL/m^2^	26.4 (14, 35)	35.5 (14.2, 62.2)	37.8 (13.9, 137.8)	29.7 (13.9, 137.8)	< 0.001

### The association between HF stage and the risk of HFH, IHD, stroke, and all-cause death

**Fig. 1:**
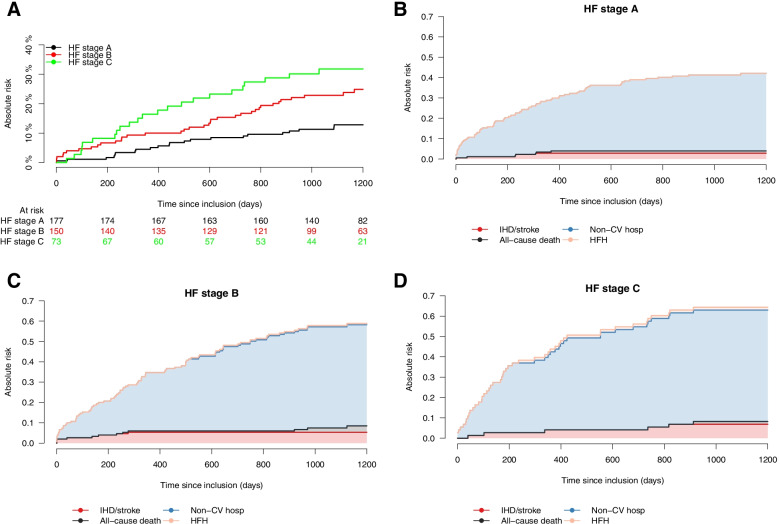
**a** Cumulative incidence of the composite of HFH, IHD, stroke and death from all causes by HF stage. Total number of events were 83. HFH, heart failure hospitalization; IHD, ischemic heart disease. **b-d** Stacked cumulative incidence plot of the risk of experiencing HFH, IHD/stroke, non-CV hospitalizations or all-cause death as the first event for (**b**) HF stage A, (**c**) HF stage B and (**d**) HF stage C. The size of each colored area represents the risk of the event. HFH, heart failure hospitalization; Hosp, hospitalization; IHD, ischemic heart disease; Non-CV, non-cardiovascular

The absolute 1-year and 3-year risks of the main composite outcome of HFH, IHD, stroke, and all-cause death are illustrated in Fig. 1a. Total number of events were 83. Within the first year of follow-up 4.5% (95% confidence interval (CI) 1.5–7.6), 10.0% (95% CI 5.2–14.8) and 16.4% (95% CI 7.9–24.9) experienced the outcome in HF stage A, B, and C, respectively. The 3-year risk of the main composite outcome was 12.8% (95% CI 7.8–17.9) in patients with HF stage A, 22.8% (95% CI 16.1–29.6) in patients with HF stage B, and 31.8% (95% CI 21.0–42.6) in patients with HF stage C. An age and sex adjusted Cox proportional hazard model yielded a significant increased hazard rate (HR) of the composite outcome in patients with HF stage B (HR: 1.46, 95% CI 0.85–2.51) and HF stage C (HR: 2.01, 95% CI 1.10–3.66) compared to patients with HF stage A (supplementary table S4).

Cumulative incidence curves of individual outcomes of HFH, IHD, stroke, and all-cause death are presented in supplementary fig. 1a-d. The 3-year risk of HFH was 10.0% (95% CI 2.8–16.3) among patients with HF stage C (supplementary fig. 1a)*.*

### Cardiovascular versus non-cardiovascular first-time hospitalizations

**Fig. 2:**
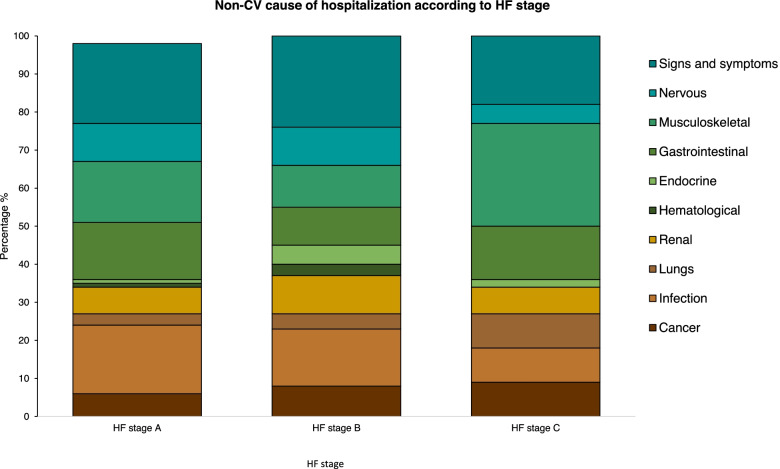
Stacked bar plot of the cause of first non-CV hospitalization in percentage by HF stage. Colors represent cause of non-CV hospitalization in legend text. Total number of non-CV events were 190. Non-CV, non-cardiovascular

The risk of non-CV hospitalization was significantly higher when compared to the combined risk of HFH, atherosclerotic events (IHD/stroke), or all-cause death in all HF stages (Fig. 1b-d). The risk of non-CV hospitalization increased with severity of HF stage and the absolute 3-year risk was 37.3% (95% CI 30.2–44.4), 49.7% (95% CI 41.6–57.8) and 54.8% (95% CI 43.4–66.2) for patients with HF stage A, B, and C, respectively. The risk of HFH was the lowest out of all categories, still patients with HF stage C had an increased risk when compared to patients with HF stage A and B. Main causes for first non-CV hospitalization were for infections, gastrointestinal, musculoskeletal, and signs and symptoms (i.e., referral codes or codes of signs and symptoms) (Fig. 2 and supplementary table S5)*.*

**Fig. 3:**
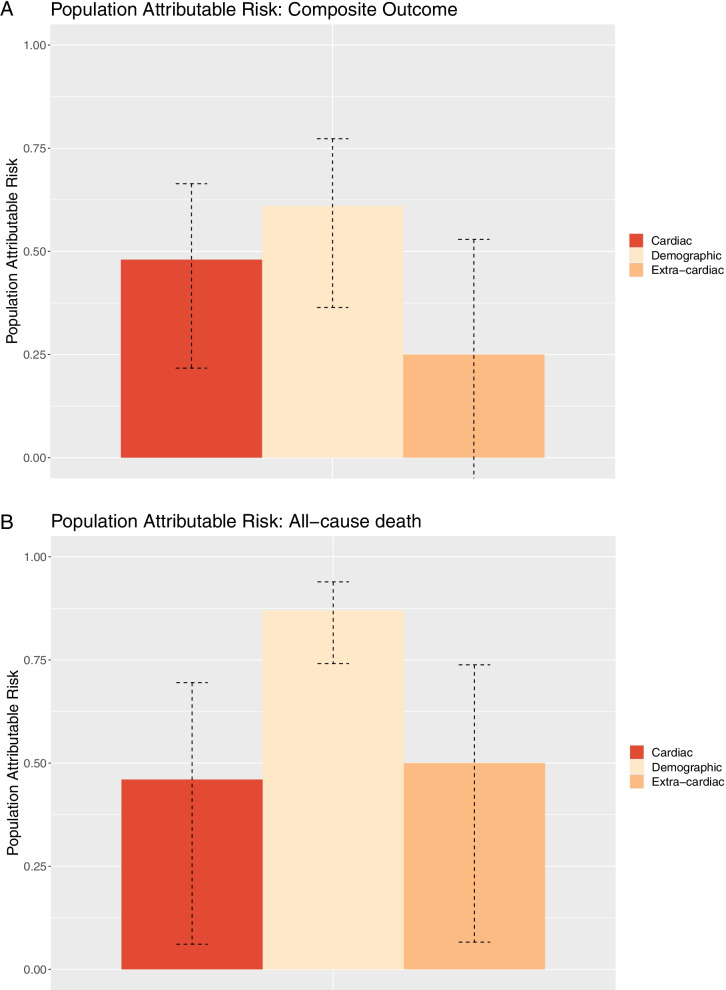
**a-b** Population attributable risk for contribution of the demographic factors, cardiac, and extra-cardiac disease burden to the outcomes of (**a**) a composite of HFH, IHD, stroke, and death and (**b**) All-cause death for all patients in the study cohort. HFH, heart failure hospitalization; IHD, ischemic heart disease.

### Relative contribution of the demographic factors, cardiac, and extra-cardiac disease burden to cardiovascular outcomes and death

Baseline risk factors were used to calculate the MAGGIC score. The mean MAGGIC score was 16.0 (SD 4.97), 18.5 (SD 5.10), 19.0 (SD 5.58) in HF stage A, B, and C, respectively. Distribution of the PAR of the main composite outcome and the outcome of all-cause death according to the demographic, cardiac, and extra-cardiac risk factors is shown *in *Fig. 3a-b. For the main composite outcome, demographic risk factors and cardiac risk factors had a PAR of 0.61 (95% CI 0.36–0.77) and 0.48 (95% CI 0.22–0.66), respectively. The extra-cardiac risk factor had a PAR of 0.25 (95% CI -0.17–0.53). No statistically significant difference between the components was observed. Regarding the outcome of all-cause death, the demographic component (PAR: 0.87 (95% CI 0.74–0.94)) contributed significantly to the greatest risk of death. There was no significant difference in the contribution of the cardiac (PAR 0.46 (95% CI 0.06–0.67)) and extra-cardiac disease burden (PAR 0.50 (95% CI 0.07–0.74)) to the risk of experiencing death.

## Discussion

### Main findings

This cohort study, including 400 patients with HF stages A-C, yielded three major findings. First, there was a gradient of risk such that patient with higher HF stage experienced more events and 10% of patients in HF stage C were hospitalized with newly detected HF within 3 years of follow-up. Second, the PAR was higher for the demographic than cardiac and extra-cardiac disease burden and contributed significantly to the risk of death. Third, when comparing the risk of HFH with non-CV hospitalizations, atherosclerotic events (IHD/stroke), and all-cause death as the first event, the risk of non-CV hospitalizations dominated regardless of HF stage.

### Other studies

Our study is complementary to three previous large observational cohort studies [1–3]. In the study by Shah et al. a worse HF stage was associated with a higher risk of death or incident HFH [3], and Xanthakis et al. also reported increasing incidence rates of HF across HF stages [1]. In our study, the absolute 3-year risk for HFH was approximately 1%, 3%, and 10% for patients with HF stage A, B, and C, respectively (supplementary fig. 1a). These results correlate well with results from Shah et al. where the 2-year risk was approximately 2%, 6%, and 11% for patients with HF stage A, B, and C, respectively, for a composite outcome of HFH or death [3] and supports previous findings regarding HFH and increasing risk with higher HF stage [1, 3]. Patients from the study by Shah et al [3]. were also elderly patients with a similar history of comorbidities as our cohort. However, in contrast to our study the follow-up time was distinctly shorter (median 608 days).

Some differences between the present and previous studies should be noted. Contrary to previous studies where the outcome of interest was survival [1–3] and incident HF [1, 3] our main outcome was a composite of HFH, IHD, stroke, and all-cause death as well as hospitalizations for non-CV causes. To our knowledge, this is the first study to evaluate the course and progression of CV and non-CV comorbidities in an elderly cohort with different HF stages. Additionally, there were differences between the present and previous studies in the outcome definition of HF. In previous studies, the outcome incident HF was defined as HFH or HF death according to ICD codes [3] or based on the Framingham criteria [1]. In the present study, HF events were defined as HFH with at least one overnight stay, thereby selecting the most severe cases of HF. HF data obtained through Statistics Denmark have previously been validated and the estimated PPV of first-time HF is 76% [14]. The patients in our study had no history of HF nor any current suspicion of HF by the treating physician compared to the participants in the Framingham Study, where patients with a history of HF were included [1]. This difference in outcome definition would likely affect the observed cases of HFH in our cohort.

### HF stages and future cardiovascular and non-CV outcomes

Our study showed a gradient in the HF stages regarding CV and non-CV outcomes in elderly patients. HF stage C was associated with the highest risk of a composite of HFH, IHD, stroke, and all-cause death and had a twofold increased rate compared to patients in HF stage A. The relative distribution and weight of the demographic, cardiac, and extra-cardiac disease burden was further illustrated by the PAR, showing an increased demographic burden amongst patients with early stages of HF to the main composite outcome and all-cause death. Our findings are similar to those found in the study by Wolsk et al.[5] where the demographic factors contributed significantly to the greatest risk of death and stroke in patients with higher LVEF.

However, the most striking observation to emerge from this study was the burden of non-CV hospitalizations, especially for conditions which cannot be interpreted as HF such as infections, gastrointestinal, and musculoskeletal, which has not previously been reported. Xanthakis et al. reported an increased non-CV mortality burden only among patients in HF stage B [1]. Previous studies have found a high prevalence of non-CV comorbidities among older patients with chronic HF [18–20] and these have been associated with adverse clinical outcomes especially in HF patients with preserved ejection fraction (HFpEF) [20]. Similarly, Ather et al. reported that patients with HFpEF had a higher non-cardiac comorbidity burden associated with higher non-HF hospitalizations compared to HFH [19]. Hence, optimal management of comorbidities may improve prognosis in HFpEF patients [19]. Our results correlate well with the findings from the EMPEROR-Preserved trial, where only a substantial part of patients experienced a HFH compared with hospitalizations for any cause [21]. Two recent studies have been published on the development and progression of HF in cohorts with a wide age range including younger adults. In a cohort consisting of 2473 participants with no history of HF, the STAAB-study reported age, female sex, and number of risk factors to be the most important determinants of structural heart disease (stage B) [22]. In slight contrast to these previous findings, our results showed no significant differences in the risk of experiencing a composite cardiac outcome for men compared to women in an older, more comorbid cohort. Similar to our findings, the MyoVasc-study reported prevalent HF in subjects who were older, often male, with a higher prevalence of comorbidities and higher intake of medication, examined in a younger cohort (age 35–84) consisting of all HF stages [23]. This underscores the heterogeneity in the development and progressing of HF in different age groups with different phenotypes of HF, highlighting the lack of a clear disease definition. Our findings show that elderly patients with early HF stages, regardless of HF stage, are more likely to experience a non-CV hospitalization as their first event, highlighting the burden of comorbidities among these patients. Our results reflect, that patients with early stages of HF are more likely to be affected by aging and their comorbid burden than HF and ischemic disease worsening.

### Strengths and limitations

This study has several strengths that should be mentioned. HF stages were defined and described accurately according to the ACC/AHA definition and no patients were lost to follow-up, enabling complete follow-up on all patients. Patients in this study were accurately included with comprehensive clinical information regarding all patients which is uncommon in most clinical registries. In addition, patients in this study had no history of HF nor any current suspicion of HF by the treating physician. Furthermore, all registries used in this study contained complete data on all patients and has previously been validated and proven to be reliable [13, 24, 25]. Some limitations of this analysis should be noted as well. This cohort represents an elderly population followed or recently admitted at a hospital, and therefore, the findings in this study do not reflect the general population. Instead, as intended, the study examines the risk of future CV and non-CV outcomes in a high-risk population, selected and included from the out-patient clinics at hospitals. Our cohort consisted of 400 patients limiting the power of reported outcomes. In addition, it was not possible to investigate important subgroups within the cohort and to adjust for additional confounders in the Cox proportional hazards model due to the sample size. An effect of misclassification cannot be excluded due to the PPV value of HF in the registries used in this study, and thus potentially affect the reported absolute risks. As a final remark, it should be mentioned that an effect of unmeasured confounders cannot be totally excluded. This could be due to the lack of paraclinical and echocardiographic examinations during the follow-up in this study; as well as an effect of volunteer bias, as healthier patients might have been more inclined to participate*.*

### Clinical perspectives

Based on our findings, a greater focus on the recognition and treatment of comorbidities in elderly patients with HF stage A-C appears warranted. Patients were followed to examine the natural course of HF in a high-risk population. However, the 3-year risk of experiencing a HFH was surprisingly low compared to the risk of non-CV hospitalizations. The adverse impact of non-CV comorbidities on future outcomes in elderly individuals with HF stages A-C appears especially profound. Our findings therefore further emphasize the importance of addressing underlying comorbidities in elderly patients with high risk for HF and early stages of HF.

### Conclusion

The risk of experiencing a composite of HFH, IHD, stroke, and all-caused death increased with higher HF stage. The PAR of the demographic burden was the highest and contributed significantly to the greatest risk of all-cause death. Furthermore, the 3-year absolute risk of experiencing HFH was 10% among patients with HF stage C. Finally, the risk of non-CV hospitalizations was the highest in all HF stages compared to the risk of HFH, stroke/IHD, and all-cause death and increased with higher HF stage.

## Supplementary Information

**Additional file 1: Table S1.** Inclusion and exclusion criteria for study population. **Table S2.** Definition of abnormal echocardiographic parameters in the Copenhagen Heart Failure Risk Study. Abbreviations: LVEF, left ventricle ejection fraction; LV, left ventricle; LVEDV, left ventricle end-diastolic diameter volume; e’, myocardial peak early velocity; E, peak velocity of early mitral inflow; GLS, global longitudinal strain. **Table S3.** ICD codes used for outcomes and procedure codes. CABG, coronary artery bypass grafting; IHD, ischemic heart disease; PCI, percutaneous coronary intervention; ICD-10, 10th revision of the International Classification of Diseases system. **Figure S1.** a-d: Cumulative incidence curves for the individual outcomes of (a) HFH, (b) all-cause death, (c) stroke, and (d) IHD by HF stage. Total number of events were HFH: 14, IHD: 22, stroke: 18, all cause death: 29. HFH, heart failure hospitalization; IHD, Ischemic heart disease. **Table S4.** A Cox proportional-hazards model adjusted for age and gender to estimate the hazard rate ratio of the composite outcome. Results presented with 95% confidence intervals and *p*-value. **Table S5.** Cause of first non-CV hospitalization by HF stage in numbers and percentages by HF stage. Non-CV, non-cardiovascular.

## Data Availability

The data that support the findings of this study are available from Denmark's Statistics but restrictions apply to the availability of these data, which were used under license for the current study, and so are not publicly available. Data are however available from the authors upon reasonable request and with permission of Denmark’s Statistics.

## Abbreviations

ACC: American College of Cardiology
AHA: American Heart Association
HF: Heart failure
CV: Cardiovascular
LVEF: Left ventricular ejection fraction
PAR: Population attributable risk
ICD-10: International Classification of Diseases 10^th^ revision
HFH: HF hospitalization
IHD: Ischemic heart disease
PPV: Positive predictive value
SD: Standard deviation
Q1-Q3: First and third quartiles
ANOVA: Analysis of variance
MAGGIC: Meta-Analysis Global Group in Chronic Heart Failure
HFpEF: HF with preserved ejection fraction
