# Supplementary material for: Association between early detected heart failure stages and future cardiovascular and non-cardiovascular events in the elderly (Copenhagen Heart Failure Risk Study)
Source: BMC Geriatr. 2022 Mar 21;22:230. doi: 10.1186/s12877-022-02875-1 (PMC8935831; doi:10.1186/s12877-022-02875-1)
Supplement: Supplementary file 1 — Additional file 1: Table S1. Inclusion and exclusion criteria for study population. Table S2. Definition of abnormal echocardiographic parameters in the Copenhagen Heart Failure Risk Study. Abbreviations: LVEF, left ventricle ejection fraction; LV, left ventricle; LVEDV, left ventricle end-diastolic diameter volume; e’, myocardial peak early velocity; E, peak velocity of early mitral inflow; GLS, global longitudinal strain. Table S3. ICD codes used for outcomes and procedure codes. CABG, coronary artery bypass grafting; IHD, ischemic heart disease; PCI, percutaneous coronary intervention; ICD-10, 10th revision of the International Classification of Diseases system. Figure S1. a-d: Cumulative incidence curves for the individual outcomes of (a) HFH, (b) all-cause death, (c) stroke, and (d) IHD by HF stage. Total number of events were HFH: 14, IHD: 22, stroke: 18, all cause death: 29. HFH, heart failure hospitalization; IHD, Ischemic heart disease. Table S4. A Cox proportional-hazards model adjusted for age and gender to estimate the hazard rate ratio of the composite outcome. Results presented with 95% confidence intervals and p-value. Table S5. Cause of first non-CV hospitalization by HF stage in numbers and percentages by HF stage. Non-CV, non-cardiovascular. [file 12877_2022_2875_MOESM1_ESM.docx]

**Supplementary material**

**Supplementary table S1:** *Inclusion and exclusion criteria for study population*
Inclusion-criteria: A total of 400 patients were consecutively included: Age ≥ 60 year with one or more risk factors for developing heart failure (HF):

- Stable ischemic heart disease
- Peripheral arterial disease
- Hypertension
- History of arterial fibrillation/arterial flutter
- Diabetes
- Apoplexy cerebri
- Chronic kidney disease.

Exclusion-criteria:

- Age ≤ 60 years
- No risk factor of HF
- Previously diagnosed with HF or admitted with heart failure. Known left ventricle ejection fraction ≤ 45 %
- Current admission with congestion or dyspnoea and suspected HF by the treating clinician.
- Advanced cardiac disease (Moderate-severe valvular disease, third-degree atrioventricular block, pacemaker with >70% ventricular pace).
- Ongoing or recent cardiac disease or procedure (Cardioversion within the previous 30 days, coronary angiograph or percutaneous coronary intervention or coronary artery bypass operation within the previous 30 days, ongoing endocarditis or myocarditis, or pulmonary embolism)
- Dialysis/estimated glomerular filtration rate <15 mL/min/1.73 m^2^.
- Moderate-severe chronic obstructive pulmonary disease (global initiative for chronic obstructive lung disease stadium (GOLD) ≥2C)
- Sepsis (systemic inflammatory response system criteria) within the previous 30 days or ongoing intravenous antibiotic

|  | Echocardiographic parameters and values: |
| --- | --- |
| Abnormal systolic function | Simpson’s biplane LVEF:  Female ≤ 53 %  Male ≤ 51 % |
| LV enlargement | LVEDV indexed to body surface area:  Female > 61 ml/m^2^  Male > 74 ml/m^2^ |
| LV hypertrophy | LV mass indexed to body surface area:  Female >96 g/m^2^  Male >116 g/m^2^ |
| Abnormal diastolic function | e’ septal:  Female < 4.1 cm/s  Male < 4.3 cm/s  Lateral E/e′ ratio > 13 or Septal E/e′ > 15 |
| Left atrial enlargement | Left atrial volume indexed to body surface:  ≥35 ml/m^2^ |
| Global Longitudinal Strain | Study definition of abnormal GLS:  Female > -15.3 %  Male > -14.7 % |
| Significant valve disease | Moderate-severe Aortic Stenosis – aortic valve area < 1cm^2^  Moderate-severe Aortic Regurgitation - visual judgment  Severe Mitral Stenosis - PG mean > 12mmHg |

**Supplementary Table S2***: Definition of abnormal echocardiographic parameters in the Copenhagen Heart Failure Risk Study. Abbreviations: LVEF, left ventricle ejection fraction; LV, left ventricle; LVEDV, left ventricle end-diastolic diameter volume; e’, myocardial peak early velocity; E, peak velocity of early mitral inflow; GLS, global longitudinal strain.*

|  | **Details on definition** | **ICD-10 and procedure codes** |
| --- | --- | --- |
| **Outcome** | | |
| Heart failure hospitalization | Defined as an overnight stay with a discharge diagnosis codes including heart failure, cardiomyopathies, hypertensive heart failure. | ICD-10: I110, I130, I132, I42, I426-29, I50 |
| Fatal and non-fatal ischemic heart disease | Defined from diagnosis and procedural codes  A fatal event was defined as having a diagnosis of myocardial infarction in the underlying cause of death. | ICD-10: I21-22, I20  CABG: KFNA, KFNB, KFNC, KFND, KFNE  PCI: KFNG02, KFNG05, KFNG10, KFNG12, KFNG02A, KFNG05A |
| Fatal and non-fatal stroke | Defined from diagnosis  A fatal event was defined as having a diagnosis of stroke in the underlying cause of death. | ICD-10: I60-64 |
| Non-cardiovascular | Defined from diagnosis codes | ICD-10: other than I01-99 |

**Supplementary table S3:** *ICD codes used for outcomes and procedure codes. CABG, coronary artery bypass grafting; IHD,* *ischemic heart disease; PCI, percutaneous coronary intervention; ICD-10, 10^th^ revision of the International Classification of Diseases system.*


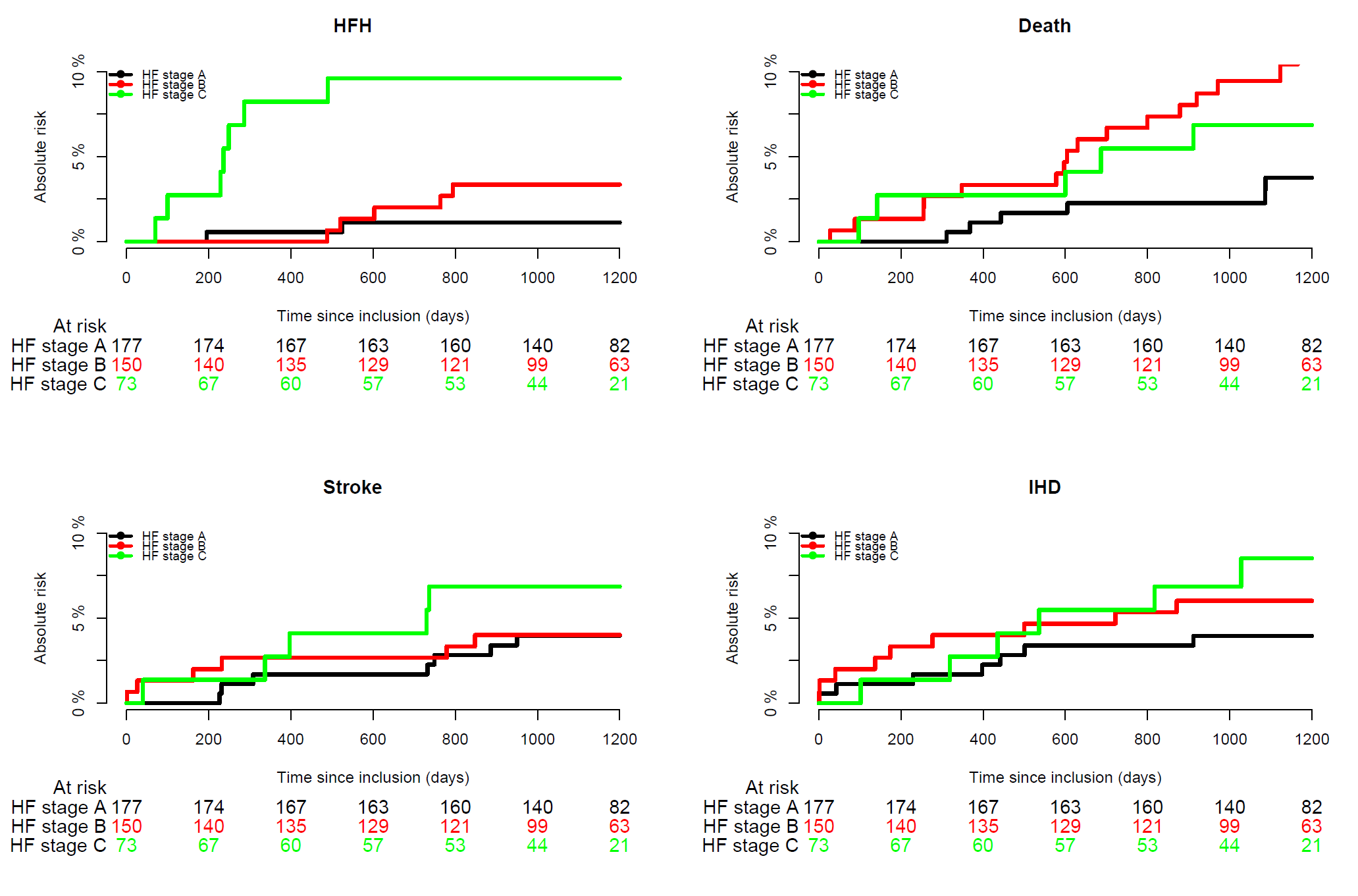


**Supplementary figure 1a-d:** *Cumulative incidence curves for the individual outcomes of* ***(a)*** *HFH,* ***(b)*** *all-cause death,* ***(c)*** *stroke, and* ***(d)*** *IHD by HF stage. Total number of events were HFH: 14, IHD: 22, stroke: 18, all cause death: 29. HFH, heart failure hospitalization; IHD, Ischemic heart disease.*

| **Variables** | **Hazard ratio** | | **95 % CI** | **P-value** |
| --- | --- | --- | --- | --- |
| **HF stage** | A | Ref |  |  |
|  | B | 1.46 | [0.85;2.51] | 0.1661 |
|  | C | 2.01 | [1.10;3.66] | 0.0224 |
| **Sex** | Female | Ref |  |  |
|  | Male | 1.34 | [0.86;2.10] | 0.2006 |
| **Age** |  | 1.06 | [1.03;1.09] | <0.001 |

**Supplementary table S4:** *A Cox proportional-hazards model adjusted for age and gender to estimate the hazard rate ratio of the composite outcome. Results presented with 95% confidence intervals and p-value.*

| **Non-CV cause** | **HF stage A**  **n (%)** | **HF stage B** | **HF stage C** |
| --- | --- | --- | --- |
| Cancer | 4 (6 %) | 6 (8 %) | 4 (9 %) |
| Infection | 12 (18 %) | 12 (15 %) | 4 (9 %) |
| Lungs | 2 (3 %) | 3 (4 %) | 4 (9 %) |
| Renal | 5 (7 %) | 8 (10 %) | 3 (7 %) |
| Hematological | 1 (1 %) | 2 (3 %) | 0 (0 %) |
| Endocrine | 1 (1 %) | 4 (5 %) | 1 (2 %) |
| Gastrointestinal | 10 (15 %) | 8 (10 %) | 6 (14 %) |
| Musculoskeletal | 11 (16 %) | 9 (11 %) | 12 (27 %) |
| Nervous | 7 (10 %) | 8 (10 %) | 2 (5 %) |
| Signs and symptoms | 14 (21 %) | 19 (24 %) | 8 (18 %) |
| **Total** | **67 (100 %)** | **79 (100%)** | **44 (100 %)** |

**Supplementary table S5:** *Cause of first non-CV hospitalization by HF stage in numbers and percentages by HF stage. Non-CV, non-cardiovascular*.
